# Changes in Types of Drinking Behavior in Korean Adults: Differences in Demographics, Depression, and Suicidal Thoughts

**DOI:** 10.3390/ijerph18147514

**Published:** 2021-07-14

**Authors:** Hye-Gyeong Son, Kyu-Hyoung Jeong, Heeran J. Cho, Minuk Lee

**Affiliations:** 1 College of Nursing, Kosin University, Busan 49104, Korea; hkprin@kosin.ac.kr; 2 Department of Social Welfare, Semyung University, Jecheon 27136, Korea; 3 Department of Health Administration, Yonsei University, Seoul 03021, Korea; 4 Mirea Social Science Institute, Seoul 07640, Korea; statmu@naver.com

**Keywords:** drinking behavior, drinking pattern, growth mixture modeling (GMM), depression, typological study

## Abstract

**Background:** Longitudinal studies of drinking behavior have reported inconsistent changes in drinking behavior as people age. Thus, this study aims to characterize the changes in drinking behavior among Korean adults and to reveal differences in their demographics, depression, and suicidal thoughts. **Methods:** This study used the Korea Welfare Panel Study data over nine years (2009 to 2017), analyzing a total of 7506 participants. Growth mixture modeling was applied to classify patterns of change in drinking in these participants. The χ^2^ test and analysis of variance were used to analyze the differences in demographics, depression, and suicidal thoughts according to patterns of change in drinking. **Results:** Changes in drinking among Korean adults were categorized into four types: “high-risk retention”, “medium-risk to high-risk”, “high-risk to low-risk”, and “low-risk retention”. Gender, age, education, marital status, living arrangement, living area, and depression differed among these groups. **Conclusion**: We identified four types of changes in adult drinking behavior in South Korea, which varied in their demographics and depression levels. These results suggest that tailoring interventions to the type of behavioral changes might be more useful than batch interventions.

## 1. Introduction

Alcohol use disorder is a major contributor to the global burden of disease, and is known to be associated with about 3.3 million deaths worldwide annually [1]. In South Korea, which has the highest per capita alcohol consumption among Asian countries [2], excessive drinking is considered a serious social problem that is closely related to physical and mental illness and is highly correlated with various crimes and accidents [3,4]. Furthermore, South Korea’s lifetime prevalence of alcohol use disorder was the highest of all surveyed mental illnesses, and 58.3% of adult drinkers were high-risk drinkers [5]. To this end, many studies on the scale and severity of alcohol abuse in South Korea have been conducted, and there has been an increasing trend toward longitudinal studies investigating abuse in recent years [6,7,8]. 

While longitudinal approaches to drinking research can overcome the limitations of cross-sectional research, cross-sectional studies also have the limitation of reporting inconsistent changes in drinking patterns. For instance, some studies have shown that drinking behavior tends to decrease as people get older [6,7], whereas other studies have shown that drinking behavior increases with age [8,9]. 

According to the Korean Survey on Mental Illness, the monthly amount of alcohol being consumed by Korean adults has been on the rise over the past few years [5]. However, it seems premature to place all adults into a singular group without a clear rationale. This treatment precludes the design of more specific interventions. 

Given the inconsistencies in past research, it is necessary to explore changes in drinking behavior using panel data with a larger sample and more sophisticated methods. We used growth mixture modeling (GMM), which identifies distinct subgroups of growth trajectories and allows previously undetected subgroups to be revealed and isolated from the main sample population. These subgroups can then be visualized for analysis by plotting each of their subgroup-specific mean trajectories, classifying changes in drinking behavior as assessed with the Alcohol Use Disorder Identification Test (AUDIT) using data from the Korea Welfare Panel Study (KOWEPS) collected between 2009 and 2017. This combined growth model allowed us to observe different types of changes in drinking behavior over time. 

In addition, this study explores differences in demographics, depression, and suicidal thoughts according to the type of change in drinking behavior. As drinking behavior is reported to be different based on demographics [10] and correlated with depression [11] and suicidal thoughts [12], examining the types of drinking changes by demographics and relating them with depression and suicidal thoughts would further enrich this study and benefit the area of research. 

The purposes of this study were as follows: (1) to identify the types of changes in drinking behavior among Korean adults, (2) to analyze the differences in demographics, and (3) to identify the relations between the drinking behavior types with depression levels and suicidal thoughts. 

## 2. Method

We utilized nine years of data from the KOWEPS between 2009 and 2017 to derive an appropriate GMM for Korean adults’ types of drinking behavior changes, and utilized the data of 2017 for demographics, depression levels, and suicidal thoughts. These allowed us to derive the appropriate number of distinct groups according to our GMM, and to interpret them in relation with demographics, depression level, and suicidal thoughts.

### 2.1. Study Participants 

The KOWEPS covered a panel of 7072 households selected from the 2005 census, and was designed to explore the current annual income, economic activities, and well-being of households and their members. It has been conducted annually since 2006. The panel covers a wide range of areas and is nationally representative. In this study, we included adults over 20 years old, as that is the legal drinking age in Korea. After excluding individuals with missing data, we ultimately analyzed data from 7506 individuals; during the study, 2.0% of the sample population passed away by committing suicide and were excluded in the data accordingly. 

### 2.2. Measurements

#### 2.2.1. Audit

The KOWEPS uses the AUDIT to measure drinking behavior; the AUDIT was designed by the WHO and comprises 10 self-report questions on harmful drinking behavior, alcohol dependence, and high-risk drinking [13]. According to the instructions in the user guide, we standardized the rating scale for all questions to range from 0 to 4 points to ensure consistency in response scores. The sum of the item scores was applied in the analysis; the AUDIT total score ranges from 0 to 40 accordingly, with higher scores indicating higher-risk drinking behavior. Less than 8 points (pts) indicates low-risk drinking, 8–15 pts likely hazardous drinking, 16–19 pts harmful drinking, and 20 pts or more a likely alcohol addiction. We used data from all nine applicable years of the KOWEPS for analysis.

#### 2.2.2. 11-Item Center for Epidemiological Studies Depression Scale (CES-D-11) 

We employed a condensed 11-item version of the CES-D-11, which was created by Kohout et al. [14] after the original CES-D scale [15]. Each item is rated on a scale of 1 (extremely rare) to 4 (almost constant) in relation to subjects′ status over the past week. We used the sum of the scores (range: 11 to 44) in the analysis, with higher scores indicating higher levels of depression. We used the KOWEPS in 2017, the twelfth wave for analysis. 

#### 2.2.3. Demographics 

We evaluated gender, age, education level, marital status, living arrangement (alone or with others), income, and region as our target demographics. Of these, income was defined as the current annual income (in 10,000s of Korean Won). For the analysis, we log-transformed the income values to ensure that the normal distribution assumption was upheld. The target region was divided into urban and rural areas.

#### 2.2.4. Suicidal Thoughts 

We evaluated suicidal thoughts using a single question with a binary response option: “I have thought about suicide” and “I have not thought about suicide”. We used the KOWEPS in 2017 for this analysis. 

### 2.3. Statistical Analyses 

We used SPSS 22.0 and M-plus 8.0. First, we described the demographics and main variables via frequency analysis and descriptive statistics. Second, we used GMM, which can isolate previously undetected subgroups from the sample population, to identify types of drinking changes over the 9 years of KOWEPS data. To assess model fit in the GMM, we used the Akaike information criterion (AIC; [16]), Bayesian information criterion (BIC; [17]), and sample-size adjusted BIC (SSABIC; [18]). We also used the entropy value and Vuong–Lo–Mendell–Rubin likelihood ratio test (VLMR test) [19]. When the values for the AIC, BIC, and SSABIC are smaller, the entropy is closer to 1 and the VLMR test is statistically significant, the model is deemed to have a more satisfactory fit to the data. Fourth, the χ^2^ test and ANOVA with Scheffe’s test were conducted to analyze differences in demographics, depression level, and suicidal thoughts according to the type of change in drinking. 

## 3. Results

The sample consisted of 7506 adults (42.0% male, 58.0% female), with an average age of about 69 years in 2017. Of all participants, 151 (2.0%) died by suicide during the study period (Table 1). 

Looking at the averages and standard deviations of drinking behavior from 2009 to 2017, we found that the data showed an overall decrease (Table 2). To verify this empirically, we used a Latent Growth Curve Model (LGCM); the model fit was satisfactory, confirming that drinking behavior decreased over time (χ^2^ = 653.287, *p* < 0.000; comparative fit index = 0.990; Tucker–Lewis index = 0.991, root mean square error of approximation = 0.045). As a linear model was found to be statistically significant in the LGCM, we tested a linear model in the GMM as well. 

The results of the model compatibility analysis of the drinking change types revealed that the fourth model had the highest AIC, BIC, and SSBIC, as well as an entropy value closest to 1, and the VLMR test was statistically significant. Consequently, classifying changes in drinking behavior into four types was the best fit for the data; the model fit is shown in Table 3.

**Figure 1:**
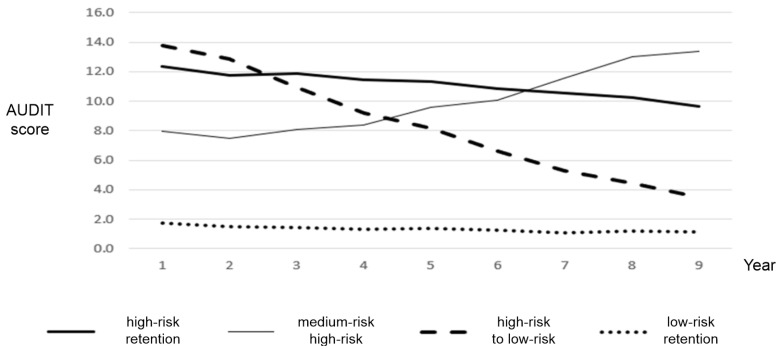
Demographics, socio-economic status, depression and suicidal ideation by types of change in AUDIT score.

Figure 1 shows the four types of changes in trajectory in AUDIT scores among Korean adults. The first type was named “high-risk retention” as it was characterized by persistently high-risk drinking behavior. This type had continued hazardous drinking from 2009 to 2017. The second type was named “medium-risk to high-risk” as these participants began with moderate drinking behavior which increased over time. The increased range over time was from 8 to 14, which indicates hazardous drinking. The third type, “high-risk to low-risk,” was characterized by a gradual decrease from high-risk drinking behavior. This type moved from hazardous drinking to low drinking. Finally, the fourth type was named “low-risk retention” as it showed persistently low-risk drinking behavior. 

Table 4 shows the differences in demographics, depression levels, and past experience with suicidal thoughts according to type of drinking change. First, for the gender differences, it was revealed that a significantly higher percentage of males were in the high-risk retention group (92.6%), medium-risk to high-risk group (81.3%), and high-risk to low-risk group (87.4%), while the low-risk retention group had a larger percentage of females (71.3%). These results suggest that there is a significant gender gap between the groups of drinkers detected by our model.

All of the differences reported between the four types of drinking behavior change groups for age, education level, marital status (spouse), living arrangement (one-person households), living area, income, and depression were statistically significant. The only differences that did not reach statistical significance were those for past experience with suicidal thoughts.

## 4. Discussion

The differences between the four groups were explored by examining the demographic factors. Males were most likely, by far, to be in the high-risk retention group and females in the low-risk retention group. These findings align with those of previous studies showing that males tend to engage in higher-risk drinking behavior and females in lower-risk drinking behavior [5]. As for age, the high-risk to low-risk and low-risk retention groups were relatively older than were the other two groups, which might reflect the characteristics of older adults, who often cannot continue their drinking behavior due to the increased presence of health problems and reduced income often associated with aging. 

More educated participants tended to belong to the high-risk retention or medium-risk to high-risk groups, whereas the low-risk retention group had a notable proportion of poorly educated individuals. People with spouses tended to fall into the high-risk retention, medium-risk to high-risk, and high-risk to low-risk groups, whereas those without spouses were more likely to belong to the low-risk retention group. The low-risk retention group had a substantial proportion of people living in single-person households, whereas the high-risk retention group primarily consisted of individuals living with others. These results do not seem to align with previous studies, many of which suggest that low education levels and insufficient family support are associated with behaviors that put people′s health at risk, such as drinking [20,21]. Furthermore, it is inferred that the reduction in drinking behavior among older adults is closely related to aging, which is associated with low levels of education, not being married, and living alone.

The high-risk retention, medium-risk to high-risk, and high-risk to low-risk groups had a higher proportion of participants living in urban areas as compared to the low-risk retention group. These results are similar to those of previous studies in which urban areas were found to have higher rates of alcohol consumption and problem drinking than rural areas [22,23]. This result seems to be derived from the fact that the proportion of workers and younger people, who consume relatively high amounts of alcohol, is higher in urban areas than in rural areas. We also found that income was relatively higher in the high-risk retention and medium-risk to high-risk groups. A lower socio-economic status has previously been found to be associated with behaviors that may put peoples′ health at risk, such as drinking and smoking [24], which does not align with our results. We attribute the relationship between income and type of drinking change in this study as reflecting the generous drinking culture of Korean society—that is, Koreans are highly permissive of drinking, and many Koreans lack awareness of the problem of alcohol abuse in Korean society. The findings indicate a need to expand anti-drinking policies to discourage drinking culture, as well as devise cultural and leisure programs that can replace drinking. 

It was also observed that depression was highest in the low-risk retention group, followed by the medium-risk to high-risk, high-risk to low-risk, and high-risk retention groups. Given that most of the people in the low-risk retention group were female, we suspect that this result relates to the fact that females generally tend to have higher levels of depression [25]. We should also note that the medium-risk to high-risk group had a relatively high level of depression. This might be explained by the fact that depression and drinking are closely related [26,27,28], so the type of increased drinking in this group might have caused their depression levels to rise over time. Therefore, intensive interventions might be required for members of the medium-risk to high-risk group with high levels of depression. 

On the other hand, while past studies have reported a statistically significant relationship between drinking and suicide [27,28], we found no such relationship. This discrepancy might be attributed to the fact that past studies considered the change in drinking to be uniform across the sample. Nevertheless, altered drinking might not offer useful insights regarding suicide interventions.

## 5. Conclusions and Implications

Regular prolific alcohol consumption is widespread in South Korean society. Drinking in groups is a regular occurrence in casual social settings, as well as events related to work such as work dinners and team-building exercises, which are both quite common and all but mandatory in the Korean workplace. Furthermore, in South Korea, most forms of alcohol are quite affordable, making drinking easily accessible to people of relatively modest means such as students, retired people on fixed incomes, and people with low incomes. As changes in Korean adult drinking behavior show four distinct types which vary in their demographics and depression levels, tailored interventions might be more useful than batch interventions to combat alcohol abuse. Given that people start drinking, and continue to drink, due to many intrinsic and extrinsic motivations, it makes sense for public interventions to have a multi-pronged approach for the greatest chance of success. This suggests that decision-makers should develop policy-driven interventions in more targeted and prudent ways to handle problem drinking, being sensitive to the social pressures and drinking culture that promote alcohol consumption in the first place.

## 6. Limitations

As this study was carried out on secondary data, it was hard to identify other factors related to drinking. There are some differences between evaluating alcohol use problems using the addiction-related symptoms (AUDIT) and the amounts/patterns of alcohol use. Therefore, in future research, it would be important to consider both the addiction scale and the alcohol use patterns. The AUDIT has different cutoffs for males and females. There are challenges in assessing severity with the same AUDIT criteria for both males and females. Participants in this study included both those who used alcohol and those who did not use it at all. Subsequent studies should explore potential differences in family history, development processes, and traumatic experiences. While this study analyzed the prevalence of alcohol consumption and the various patterns of alcohol consumption across South Korea, there are several demographics that are not accounted for. For example, although our model examines the trajectory of drinking behavior in great depth, we have not yet seriously considered how other available factors impact drinking. For example, we have further information about living arrangements and income, but we do not have access to data about what brought about these various living situations. To make a more complete model that can take into account the various motivations and living situations of alcohol consumers, it will be necessary to gather more extensive data in the forms of in-person interviews or surveys. This will require a considerable amount of resources, but it would allow us to make the best predictions as to what types of interventions will be the most efficient and effective in relieving the high rate of problem drinking in South Korea.

## Figures and Tables

**Table 1:** Demographics of survey participants.

Variables		2017 (*n* = 7506)
		*n* (%)
Gender	Male	3149 (42.0)
	Female	4357 (58.0)
Age	≤39	155 (2.1)
	40–49	812 (10.8)
	50–59	1468 (19.6)
	60–69	1338 (17.8)
	70–79	1297 (17.3)
	≥80	2436 (32.5)
Education	Less than elementary school	2365 (31.5)
	Middle school	998 (13.3)
	High school	2215 (29.5)
	University or higher	1928 (25.7)
Spouse	Yes	5139 (68.5)
	No	2367 (31.5)
One-person households	Yes	1327 (17.7)
	No	6179 (82.3)
Area	Urban	5809 (77.4)
	Rural	1697 (22.6)
Income	M = 4841.81 (SD = 4282.63)	

M = mean; SD = standard deviation.

**Table 2:** Means and standard deviations of the AUDIT scores between 2009 and 2017.

	2009	2010	2011	2012	2013	2014	2015	2016	2017
M	3.92	3.56	3.48	3.26	3.29	3.10	2.96	3.03	2.89
SD	5.56	5.21	5.18	5.05	4.97	4.95	4.86	4.88	4.73

M = mean; SD = standard deviation.

**Table 3:** Fitness of the models of change types in AUDIT score.

Class	Model Fit						
	LL	AIC	BIC	SSABIC	Entropy	VLMR *p*-Value	*n* (%)
1	175,842.867	351,713.734	351,810.663	351,766.173			
2	174,063.248	348,160.496	348,278.195	348,224.173	0.923	0.000	1350 (18.0)
							6156 (82.0)
3	172,975.289	345,990.579	346,129.048	346,065.492	0.942	0.000	5738 (76.45)
							1355 (18.05)
							413 (5.50)
4	172,374.601	344,795.201	344,954.441	344,881.352	0.932	0.001	745 (9.93)
							486 (6.48)
							445 (5.93)
							5830 (77.67)
5	171,984.487	344,020.974	344,200.984	344,118.361	0.929	0.207	659 (8.78)
							223 (2.97)
							5588 (74.45)
							461 (6.14)
							575 (7.66)

LL = loglikelihood; AIC = Akaike information criterion; BIC = Bayesian information criterion; SSSABIC = sample-size adjusted BIC.

**Table 4:** Differences in demographics according to type of change in AUDIT scores variables.

Variables		1. High-Risk Retention ^a^	2. Medium-Risk to High-Risk ^b^	3. High-Risk to Low-Risk ^c^	4. Low-Risk Retention ^d^	χ^2^/F/ Difference (Scheffe)
		*n* (%)	*n* (%)	*n* (%)	*n* (%)	
Gender	Male	690 (92.6)	395 (81.3)	389 (87.4)	1675 (28.7)	1890.086 ***
	Female	55 (7.4)	91 (18.7)	56 (12.6)	4155 (71.3)	
Age	Mean (SD)	61.83 (12.24) ^a^	59.45 (12.46) ^b^	69.58 (14.29) ^c^	70.93 (16.35) ^d^	142.040 *** b < a < c,d
Education	Less than elementary school	81 (10.9)	43 (8.8)	106 (23.8)	2135 (36.6)	404.744 ***
	Middle school	83 (11.1)	52 (10.7)	70 (15.7)	793 (13.6)	
	High school	319 (42.8)	211 (43.4)	139 (31.2)	1546 (26.5)	
	University or higher	262 (35.2)	180 (37.0)	130 (29.2)	1356 (23.3)	
Spouse	Yes	577 (77.4)	368 (75.7)	357 (80.2)	3837 (65.8)	87.173 ***
	No	168 (22.6)	118 (24.3)	88 (19.8)	1993 (34.2)	
One-person households	Yes	67 (9.0)	45 (9.3)	47 (10.6)	1168 (20.0)	100.003 ***
	No	678 (91.0)	441 (90.7)	398 (89.4)	4662 (80.0)	
Area	Urban	641 (82.4)	398 (81.9)	349 (78.4)	4448 (76.3)	20.657 ***
	Rural	131 (17.6)	88 (18.1)	96 (21.6)	1382 (23.7)	
Income	Mean (SD)	6307.14 (4257.98) ^a^	6274.32 (3906.16) ^b^	4764.76 (3627.15) ^c^	4541.02 (4300.25) ^d^	58.130 *** a,b > c,d
Depression	Mean (SD)	13.07 (3.20) ^a^	14.13 (4.45) ^b^	13.96 (4.19) ^c^	14.73(4.93) ^d^	30.573 *** a < b,c < d
Suicidal ideation	Yes	7 (0.9)	11 (2.3)	8 (1.8)	125 (2.1)	5.122
	No	738 (99.1)	475 (97.7)	437 (98.2)	5705 (97.9)	

*** *p* < 0.001.

## Data Availability

The data that support the findings of this study are available from Korea Welfare Panel Study upon request at https://www.koweps.re.kr:442/eng/data/data/list.do (accessed on 1 July 2019).
